# Modelling local field potential features during network gamma oscillations

**DOI:** 10.1186/1471-2202-15-S1-P131

**Published:** 2014-07-21

**Authors:** Richard J Tomsett, Matt Ainsworth, Alexander Thiele, Mehdi Sanayei, Xing Chen, Alwin Gieselmann, Miles A Whittington, Mark O Cunningham, Marcus Kaiser

**Affiliations:** 1 School of Computing Science, Newcastle University, NE1 7RU, UK; 2 Institute of Ageing and Health, Newcastle University, NE4 5PL, UK; 3 Hull York Medical School, University of York, YO10 5DD, UK; 4 Institute of Neuroscience, Newcastle University, NE2 4HH, UK

## 

While the physics of local field potential (LFP) generation are well-established, the complexity of neural network dynamics means that interpreting LFP measurements in terms of the underlying neural activity is very difficult [1]. Recent studies have therefore investigated forward models of the LFP: calculating the LFP due to known arrangements of neuronal current sources [1]. We use this approach to study the spatio-temporal features of the LFP recorded during persistent gamma-oscillations *in vitro*. The mechanism by which this activity regime is generated is well-known [2], and many multi-electrode array recordings are available that allow us to compare the spatio-temporal properties of the experimentally measured and simulated LFPs. For simplicity, we implemented a model of a neocortical slice containing only layer 2/3, using our VERTEX simulation tool. The model contained a gamma oscillation-generating region surrounded by regions that did not participate in the gamma oscillation (i.e. the neurons in these regions fired randomly). These enforced conditions allowed us to study the spatial profile of the LFP from differently sized gamma oscillation-generating patches, while minimising the number of variables we changed between simulations. Additionally, we could make observations about properties of the LFP during random activity in the network.

We describe three key results from our simulations. Firstly, we found that perisomatic synaptic currents on pyramidal neurons resulting from basket interneuron firing dominate the LFP during gamma oscillations, in agreement with recent experimental results [3,4]. We predict that basket interneurons will also contribute the majority of the LFP signal during random, uncorrelated activity because of the location of their synapses at pyramidal neuron somas. This contribution is amplified during oscillatory activity because of increased basket cell synchrony relative to other neuron types. Secondly, we investigated how gamma-frequency power from a localised gamma-generating region in the model spread due to volume conduction. We found that the spatial spread of the oscillation in the LFP increased above and below the neurons' soma-depth, and depended on the level of the random surround activity. Finally, we investigated frequency scaling in the LFP power spectrum across space. We showed that LFPs at the level of the pyramidal cells' apical dendrites exhibit a low-pass filtering effect (as predicted previously [5]), which is absent from LFPs recorded at the soma level because of the relative dominance and localisation of the basket cell input. We confirmed that this matched the frequency scaling in our comparison experimental data recorded in macaque neocortical slices, and, based on the frequency characteristics in our model, propose that the experimentally recorded LFP could contain contributions from spiking activity at frequencies down to, and perhaps below, 100 Hz.
